# Open Educational Resources in Behavior Analysis

**DOI:** 10.1007/s40617-019-00371-4

**Published:** 2019-08-08

**Authors:** Veronica J. Howard

**Affiliations:** Psychology Department, University of Alaska, 3211 Providence Drive, Anchorage, AK 99508 USA

**Keywords:** Access, Financial Accessibility, Higher Education, Open Educational Resources

## Abstract

Open educational resources (OERs) are materials that can be freely downloaded, edited, and shared to better serve all students. These resources are typically free of cost, reducing barriers to access for students and ensuring that all learners can have access to educational materials regardless of their financial status. OERs have been demonstrated to improve student performance and retention, especially for students traditionally underrepresented in higher education (e.g., first-generation, non-White students). Although there have been informal calls for additional OERs in behavior analysis, it is unclear whether behavior-analytic OERs exist. The aim of the current study was to use an OER aggregating metafinder to review what OERs are available on topics related to behavior analysis and whether sufficient resources exist to serve as primary course materials. Results indicate that OERs for behavior-analytic content exist but tend to be written by nonbehaviorists for use in survey courses in mainstream psychology. There also do not appear to be sufficient resources to support a course. Implications for promoting the development and dissemination of OERs, particularly with respect to increasing the recruitment and retention of diverse students in the field of behavior analysis, are discussed.

## Cost, Access, and Equity

For the great majority of our boys and girls, the kind and amount of education they may hope to attain depends, not on their own abilities, but on the family or community into which they happen to be born.

—Truman Commission (President’s Commission on Higher Education, 1947)

Recognizing the central role that access to higher education plays in economic mobility and a strong democracy, President Harry S. Truman commissioned the first ever federal analysis of the state of higher education in 1946 (The American Presidency Project, n.d.). The subsequent six volume *Higher Education for American Democracy* commission report (President’s Commission on Higher Education, 1947) highlighted major limitations of the United States’ educational system, including overcrowding and limited access to students without exceptional talent and financial resources. This report was a call to action to increase access to higher education for Americans.

To address these barriers, a series of sweeping higher education reforms were instituted. Community colleges began offering 2-year degrees and free public education for the first 2 years of college beginning in the 1940s, and continue to serve as a path or ultimate destination for postsecondary degrees for millions of Americans every year (Ginder, Kelly-Reid, & Mann, 2018). The Higher Education Act of 1965 further increased access to continued education through the advent of modern financial aid through the Pell Grant (named in honor of HEA sponsor Senator Claiborne Pell). To date, the proportion of students qualified to receive the Pell Grant remains the most commonly used proxy measure for determining the populations of low-income students at institutions of higher education (Janice & Voight, 2016). Although scholars debate whether Truman’s reform aims were ever truly achieved, particularly with respect to access (Hutcheson, 2007), the state of higher education was transformed by these federal reforms (Gilbert & Heller, 2013).

Modern higher education has become increasingly unrealistic for many students from low-income families (families whose taxable income did not exceed 150% of the federal poverty level, determined by factors such as family size and location). As of January 2019, a family of four would be designated “low-income” if the combined household income was anywhere from $38,625 in the contiguous United States to $48,285 in Alaska (US Department of Education, 2019). In 2017, Hispanic households and Black households had substantially lower median household incomes ($49,793 and $40,232, respectively) than White households ($63,704) and Asian households ($83,456; Guzman, 2018), making students from ethnic minorities disproportionally represented in this low-income family designation.

Issues of financial access are a systemic challenge to diversity in higher education. Compared to students from affluent households, students from low-income families are more likely to drop out of high school (America’s Promise, 2014), less likely to enroll in college (Carnevale, Smith, Melton, & Price, 2016), more likely to require compulsory remedial coursework during college (Radwin, Wine, Siegel, & Bryan, 2013), and are four to five times less likely to earn a 4-year degree (Lauff & Ingels, 2013; Pell Institute, 2016). Federal financial aid policies have also shifted, prioritizing scholarships and financial support for students from middle-income families, leaving students from low-income households to shoulder the burden of unmet financial need through other forms of financial aid like federal and private loans (Shapiro, Dundar, Yuan, Harrell, & Wakhungu, 2014).

To help mitigate the high cost of attendance, many low-income students choose to work through college. Research shows that working students are more likely to earn poor grades, especially as they approach full-time employment (Carnevale et al., 2016). Students from diverse backgrounds are again overrepresented in this category. Working students are more likely to be over 30 years of age, female, Black or Latino, first-generation college students, and English language learners or students who speak English as a second language (Carnevale & Smith, 2018). Students may simply choose to complete their degrees more slowly to balance work and school, but this also reveals the disparity between affluent and poor students. Part-time students are only half as likely to complete a college degree in 6 years compared to full-time students, with Black and Hispanic students disproportionately overrepresented in part-time enrollments (Shapiro et al., 2014).

The financial barriers described previously make it difficult for traditionally underrepresented populations to persist in college and earn a degree. For students entering college during 2016, 85.3% of Asian students and 78.6% of White students persist through the first year of college and reenroll during the second year, compared to only 67.0% of Black students and 70.7% of Hispanic students (NSC Research Center, 2018). This achievement gap appears to only widen over time. For students entering college during 2010, 38% of Black and 45.8% of Hispanic students had completed their 4-year degree by 2016, compared to 63.2% of Asian students and 62.0% of White students (NSC Research Center, 2017).

Taken together, these statistics on income and financial accessibility demonstrate that there are wide-spread systemic barriers for low-income and minority students pursuing higher education. One way to promote diversity within the field of behavior analysis could be to find ways to recruit and retain a more diverse group of undergraduates entering the field. Although it may not be possible for professors to directly target many of the costs associated with higher education, professors have direct control over one major hidden cost of education: required course materials and textbooks.

### Textbook Affordability and Open Educational Resources

The cost of textbooks has increased over 1,000% since 1977, far outpacing the cost of any other consumer goods or services (Bureau of Labor Statistics, n.d.). A variety of studies have shown that students report *sometimes* (up to 60%) or *regularly* (around 25%) going without their required materials due to cost (Chae & Delaney, 2018; Florida Virtual Campus, 2012, 2016). Most students report paying for some or all their textbooks out of pocket because the rising cost of tuition and fees is not being offset by increases in financial aid (Florida Virtual Campus, 2016), meaning that students must either go without required course materials or find some other way of accessing the materials (e.g., working extra hours, sharing books, or finding alternative editions of the material). These studies also indicate that textbook costs are associated with other challenges. Students report taking fewer credits, choosing classes strategically to lower textbook costs, dropping classes due to the cost of the books, or withdrawing from the course due to low grades associated with not having the required materials, meaning that financial resources are a direct barrier to student success.

To address the rising cost of textbooks, open educational resources (OERs) have swiftly gained popularity in higher education. As of 2017, OERs have been adopted in 16.5% of large introductory undergraduate courses, consistent with the use of comparable commercial textbooks (Seaman & Seaman, 2017). OERs tend to be digital and free of cost to users, meaning that students can quickly and easily download their course materials, without encountering paywalls, through repositories such as the University of Minnesota’s Open Textbook Network (2018) and Rice University’s Openstax initiative (2018).

There is no consistently used definition of OER (Wikicommons, 2016), yet most definitions focus on material (a) being free of cost to the user and (b) including permission for the user to retain, adapt, redistribute, and create derivative products (e.g., Hewlett Foundation, 2016; SPARC, 2018a). These later rights are referred to as the 5 Rs: the rights to retain access to the work indefinitely, to reuse the work in a variety of ways, to revise the content, to remix the content by combining with other material to make something new, and to redistribute the work, including any derivative versions created, to others. To better understand the permissions associated with OERs, as well as common misconceptions about the nature of open materials, it is prudent to briefly review relevant copyright licenses.

### Understanding Copyright Licenses

Traditionally speaking, copyright refers to an author’s or owner’s right to control the way the content is used, as well as any revenue generated by that content (17 U.S.C. § 102, 1990). Since the founding of the United States, works either fell under copyright law, with all rights reserved to the author, or existed in the public domain and could be used, adapted, or redistributed by anyone without permission. To use, adapt, or create a derivative work from copyrighted material, a creator would need to secure the permission of the original author.

Critics argue that copyright law has overwhelmingly favored the economic rights of the creator with little thought given to other values, such as promoting fairness and innovation, increasing the pace of scientific discovery, and forming a just and attractive culture (Fisher, 2001; Gordon, 1989; SPARC, 2018b). With the goal of improving education, the fair use doctrine (17 U.S.C. § 107, 1992) permits instructors to use copyrighted material in the classroom without first seeking the permission of the copyright holder. It is important to note that there is a variety of factors that influence whether it is acceptable to use copyrighted content for teaching (e.g., the nature of the work, the profit for the user, the financial impact to the author, the substantiality of the portion used relative to the whole of materials), and there are no specific rules describing what type or amount of use is covered by fair use doctrine. For additional advice, readers are encouraged to review the guidance provided by the US Copyright Office (2014).

Nevertheless, there was no way for creators to share some but not all permissions protected by copyright with their audience until Creative Commons licenses were created and released in 2002 (Creative Commons, n.d.). Creative Commons (hereafter referred to as CC) licenses permit all users to retain and redistribute the work. There are four additional conditions specified by these licenses: attribution (i.e., use permitted only if the author is credited for the work), share-alike (i.e., use permitted only if the licensee distributes the work under a license identical or less restrictive than the source’s license), noncommercial (i.e., use permitted only if the licensee uses the work or derivatives and does not profit from them), and no derivative works (i.e., the licensee may use the work but not make derivatives or remixes of the work). From these rights, six regularly used CC licenses were developed: Attribution/CC BY, Attribution-ShareAlike/CC BY-SA, Attribution-NonCommercial/CC BY-NC, Attribution-NonCommercial-ShareAlike/CC BY-NC-SA, Attribution-NoDerivatives/CC BY-ND, and Attribution-NonCommercial-NoDerivatives/CC BY-NC-ND (see Table 1).

**Table 1:** Copyright licenses

Specific license	Copy and publish	Attribution required	Commercial use	Modify and adapt	Change license
Public Domain / CC Zero	Yes	No	Yes	Yes	Yes
CC BY	Yes	Yes	Yes	Yes	Yes
CC BY-SA	Yes	Yes	Yes	Yes	No
CC BY-NC	Yes	Yes	No	Yes	Yes
CC BY-NC-SA	Yes	Yes	No	Yes	No
CC BY-ND	Yes	Yes	Yes	No	Yes
CC BY-NC-ND	Yes	Yes	No	No	Yes

*Note*. Overview of permissions and restrictions associated with each copyright license. Licenses, in order, include: CC BY/reuse with attribution, CC BY-SA/reuse with attribution and share derivative works alike, CC BY-NC/Reuse with attribution for noncommercial purposes, CC BY-NC-SA/ Reuse with attribution for noncommercial purposes and share derivative works alike, CC BY-ND/Reuse with attribution but create no derivative works, and CC BY-NC-ND/ Reuse with attribution for noncommercial purposes but create no derivative works. Table adapted from *Quick Guide to Creative Commons Licenses for Open Educational Resources (OER)*, work licensed as CC BY, by D’Arcy Hutchings of University of Alaska/Alaska Pacific University Consortium Library (https://libguides.consortiumlibrary.org/OER/isit)

Even though work shared under a CC license is free to access, it may not be free of copyright restrictions. The variations of CC licenses give content creators a way to communicate to users exactly what permissions they wish to share and those they do not. For example, a CC BY-NC-SA license would permit users to access, retain, and adapt the copyrighted material, but the licensee would be required to share the material under a similar or less restrictive open license and could not use the copyrighted material for commercial gain.

CC licenses are also nonexclusive and nonrevocable, meaning that any works obtained under a CC license may continue to be used under that license indefinitely (creativecommons.org, n.d.). A nonrevocable license offers great benefits to instructors and course designers because it means that OER users can—at a minimum—continue to use, retain, and redistribute the material as found perpetually and without requiring the permission of the author. By comparison, instructors seeking permission to use copyrighted material for educational purposes under fair use doctrine would have to clearly specify what material would be used, how much of the material would be used, who the material would be shared with, how long the instructor would be able to share that material with users, and so on, and permission to use the work could be revoked at any time.

Educational material shared with a CC license also helps mitigate costs associated with new editions and challenges associated with textbooks going out of print or changing publishers. For example, *Research Methods in Psychology*, an OER research methods textbook originally published with a now-defunct commercial publisher, was released under a CC license. Because of that license, a new author was able to adapt and revise that work and rerelease it under a CC license with attribution to the first author. Later, additional authors adapted the same textbook to better suit students with more diverse or specific needs. In short, CC licenses helped protect the rights of the author while permitting the robust reuse and adaptation of this textbook, resulting in four different, high-quality versions of this educational material.

### OERs and Student Success

The impact of OERs on the educational experience of students and instructors has received recent attention in scholarly research. Studies have explored topics such as student and instructor perception of the quality of open and commercial textbook materials (Bliss, Robinson, Hilton, & Wiley, 2013; Brandle et al., 2019), barriers to the adoption of OERs (Seaman & Seaman, 2017, 2018), and student performance when using OERs compared to using commercial materials (Clinton, 2018; Gurung, 2017; Jhangiani, Dastur, Le Grand, & Penner, 2018). A 2016 meta-analysis of studies on the efficacy and perception of OERs reports that most studies show favorable perceptions of the quality and effectiveness of OER products from students and faculty (Hilton, 2016). This meta-analysis also reports that students using OERs tend to do as well as or better than students in the same courses using commercial textbooks. It is unclear whether this difference is due to factors like alignment between course materials and course content or early access to educational materials (cf. Grimaldi, Basu Mallick, Waters, & Baraniuk, 2019). However, Hilton’s (2016) meta-analysis also highlighted weaknesses in the available research, including a lack of control for teacher and student effects, a lack of large-scale pre- and postcourse performance metrics, and a lack of random assignment to OER vs. commercial use conditions.

Fischer, Hilton, Robinson, and Wiley (2015) compared the academic performance of 16,727 students across 10 educational institutions to determine what effect the transition from commercial to OER textbooks had on student performance. The authors evaluated student performance in 15 courses where instructors moved from commercial textbooks to OERs, choosing student performance metrics available through institutional records, including course persistence (i.e., completing the course), earning a passing grade (C+ or higher), overall course grade, and the number of credits taken both during a semester when using an OER textbook and in the following semester. Researchers reported that students in courses using OERs were more likely to persist through the course, with 6% student withdrawals using OERs compared to 21% student withdrawals using commercial textbooks.

Fisher et al. (2015) also report that for most courses, there were no statistically significant differences in passing grades or overall course grades (9 courses and 10 courses, respectively), but performance did improve in a small set of courses (more passing grades in 5 courses and higher overall grades in 4 courses). In only one course did students perform better using commercial textbooks. There were also indirect benefits, with students using OERs enrolling in more courses during the same semester than peers using commercial products, *t*(8101) = 27.81, *p* < .01, as well as in the subsequent semester when credit loads in the prior semester were held constant, *F*(1, 6440) = 154.08, *p* < .01. Lack of disaggregated student data about factors such as information about student enrollment status (part time vs. full time) or financial need was a significant limitation of this study.

Colvard, Watson, and Park (2018) conducted a similar study, which included variables such as enrollment density and financial need that were not clearly evaluated in previous studies. Researchers compared performance, measured by final grades and grades of D, F, or W, for students taking eight courses before and after the adoption of OERs. Greater access to student performance data and institutional metrics permitted the researchers to also evaluate the impact of OER adoption on student performance specifically for (a) low-income students, (b) non-White students, and (c) part-time students. Results indicated that there was a statically significant overall improvement in final course grades when using OERs relative to using commercial textbooks, *t*(21, 820) = −15.95, *p* < .001, as well as a reduction in DFW grades by 2.68%. The gains achieved by adopting OERs disproportionately improved performance of Pell recipients, *F*(1, 21,818) = 9.348, *p* = .002, non-White students, *F*(1, 19,012) = 10.374, *p* = .001, and part-time students, *F*(1, 21,818) = 59.68, *p* < .001.

These previous studies indicate that adopting OERs improves performance, especially for students traditionally underrepresented in higher education. Increasing access to low-cost and easily accessible educational materials in behavior analysis has been called for informally (OpenBehavioralScience.org; Pavone, 2018), yet it is unclear to what extent these materials exist to be adopted. This review aims to assess what types of behavior-analytic OERs exist with special attention paid to whether the materials could be used to support a course in behavior analysis.

## A Review of Behavior Analysis OERs

To evaluate whether behavior analysis OERs exist, it was necessary to develop a method to search for available resources. Many OER repositories exist, ranging from popular sites like Merlot (2019; https://www.merlot.org/merlot/) and the Open Textbook Network (2018; https://open.umn.edu/opentextbooks) for materials geared toward educators to niche archives like the HathiTrust (n.d; https://www.hathitrust.org/) and Project Gutenberg (2019; http://www.gutenberg.org/) with primary sources and materials residing in the public domain. However, it was unclear which repository would be most appropriate to find resources that could be used to teach an introductory course in behavior analysis.

A review of the literature returned no studies critically comparing the quality of different repositories to one another on features such as consistency of search results, number of resources, quality of resources, user-friendliness, and so on. Rather than choose a single OER repository, the author selected an OER metafinder tool to search multiple OER repositories for materials related to the study of behavior analysis. What follows is a description of the method used to discover the OER resources evaluated in this project.

### Search Procedure

**Fig. 1.**
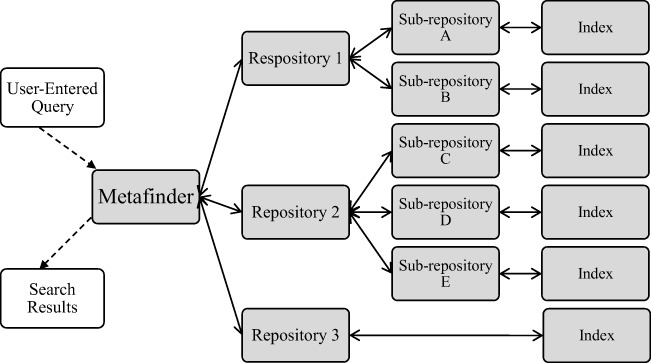
Model of federated search across three hypothetical repositories

The author searched the George Mason University Mason OER Metafinder (2018; hereafter referred to as MOM) to find materials related to the science and practice of behavior analysis. MOM is a federated search information-retrieval interface designed to aggregate search results from multiple linked repositories (Jasco, 2004). Federated searches engines provide the user with a single point of access to simultaneously search multiple repositories and their respective subrepositories, which search their indexed data for the metadata tag provided. Each subrepository then communicates results back to the querying repository, and each repository back to the metafinder, as modeled in Fig. 1. During the initial search in fall 2018, MOM interfaced with search engines for 17 major OER repositories.

Using MOM permitted an efficient one-stop query of multiple repositories. Search terms included those that may yield the most results related to the science and practice of behavior analysis, or those that may be most likely to be used by instructors developing an introductory course in behavior analysis: “behavior analysis,” “applied behavior analysis,” “operant conditioning,” and “functional behavior assessment.” Resource records returned via this search method were analyzed to determine whether the available record metadata appeared related to behavior analysis. Materials unrelated to Skinner’s behavior analysis, results with broken or inaccessible links, and duplicated search results were omitted from the analysis. This initial search yielded a total of 88 resources (17 for the term “behavior analysis,” 19 for “applied behavior analysis,” 35 for “operant conditioning,” and 17 for “functional behavior assessment”). These search results were further combined into a single set of resources, and 24 duplicate records that appeared in more than one search were omitted from the final list of resources, yielding a set of 64 unique records.

To ensure consistency of search procedures and coding protocol, the author and a research assistant independently searched MOM for each set of search terms and independently coded each resource found using the initial coding protocol described previously. At this point, the author discovered that the same search terms could yield a differing number of results on each subsequent search. MOM is a real-time, federated search engine. Unlike other aggregating search engines that preindex search results (e.g., Google), MOM connects to each of the linked repository search engines and runs a real-time duplicated series of searches using the repository’s site search syntax at the point a query is entered. Resources returned to MOM can be influenced by factors ranging from changes in repository metadata records to connectivity issues between the metafinder and each repository and index (Grotophorst, personal communication, March 20, 2019).

Nevertheless, each reviewers’ searches returned relatively consistent numbers of search results. Agreement was calculated by dividing the smaller number of search results returned by the larger, then multiplying by 100 to create a percentage. Agreement on the resources returned was 96% (range 84%–100%).

### Resource Coding

#### Category

To evaluate the usefulness of each item for the training of new behavior analysts, the author reviewed each item provided and classified the materials into major categories, including (a) applications, computer software, or programs that run on mobile devices or personal computers (e.g., data collection, clinical, or simulation programs); (b) textbooks, or full-length texts focused specifically on topics related to behavior analysis; (c) chapters, or multipage sections of a larger, full-length text focused specifically on topics related to behavior analysis; (d) course builds, or collections of materials presented as a full course preparation; (e) course elements, including videos, ancillary materials like presentation handouts, test banks, and other materials that could be used to supplement a course; (f) references, or links to theses or full-length articles on the topic of behavior analysis; and (g) websites. (See column “Resource Type” in Table 2.)

**Table 2:** George Mason University OER Metafinder (MOM) Resource Search Results

Resource type	Author and title	Source repository	License	BACB 5th Ed. task list designation
Application	Special Appucations Inc. *ABA Find It! App for iOS*	Merlot.Org	©	
Application	Marz Consulting Inc. *Behavior Tracker Pro App for iOS*	Merlot.Org	©	
Application	Special Learning Inc. *My First Voice 2 App for iOS*	Merlot.Org	©	
Application	Brain Parade. *See.Touch.Learn. App for iPad*	Merlot.Org	©	
Textbook	Sennott et al. *Comprehensive Individualized Curriculum and Instructional Design: Curriculum and Instruction for Students*	Open Textbook Library	CC BY-NC	A-5 B-4, 6^a^ F-3 ,4, 6, 7 G-4, 14^a^, 3
Textbook	Desrochers & Fallon. *Instruction in Functional Assessment*	Merlot.Org	CC BY-NC-SA	B-4, 7, 9, 11^a^, 12 C-1, 2^a^, 4, 8, 11 D-1, 5^a^ E-3 F-1^a^, 3, 5, 6, 7, 8, 9 G-1, 2, 5, 6, 14, 15 H-1, 2, 3, 4, 5, 6, 7, 9^a^
Textbook	Homme. *Laboratory Studies in the Analysis of Behavior: A Manual of Operant Conditioning Procedures for Students in Behavioral Psychology*	Digital Public Library of America	CC Zero	B-1^a^, 2^a^, 5, 11, 12^a^ C-3^a^, 4^a^, D-1 G-8
Course Element (Lecture)	Wolfe. *3: Learning: The Power of Association | Audio Lectures and Notes . . .*	MIT OpenCourseWare	CC BY-NC-SA	B-3^a,b^
Course Element (Lecture)	No author. *Behaviorism Thorndike’s Connectionism Became Refined and . . .*	MIT OpenCourseWare	Unknown	
Course Element (Study Guide)	Schneider. *Lecture 29 Notes: Konrad Lorenz on Learning*	MIT OpenCourseWare	Unknown	B-3^a^
Course Element (Study Guide)	No author. *Lecture Three: Association Learning and Behaviorism*	MIT OpenCourseWare	CC BY-NC-SA	B-3^a^
Course Element (Study Guide)	Anonymous MIT student. *Study outline for Kosslyn and Rosenberg “Introducing Psychology” 4 . . .*	MIT OpenCourseWare	CC BY-NC-SA	B-3, 4, 5^b^, 6, 8^a^, 9, 11 G-7
Course Element (Study Guide)	Roscello, A. *PSY101 – Topic 5 – Learning*	OER Commons	CC BY-NC	
Course Element (Study Guide)	No author. *Psychology, Learning*	OER Commons	CC BY-NC-SA	
Course Element (Test)	Gabrieli. *2009 Practice Exam 1 Solutions*	MIT OpenCourseWare	CC BY-NC-ND	D-1 B-5^a^, 11^a^ G-7^a^
Course Element (Video)	Hayes. *CareLog: A Selective Archiving Tool . . . CareLog: A Selective Archiving Tool for Behavior Management in Schools*	Merlot.Org	CC BY-NC-ND	
Course Element (Video)	FuseSchool. *Conditioning Animals: Learning Behaviour | Ecology & Environment | The Virtual School*	OER Commons	©	B-3^b^, 4^b^
Course Element (Video)	Khan Academy. *Khan Academy MCAT Prep – Learning*	Merlot.Org	CC BY-NC-SA	B-3^a^, 4, 5^b^, 6 G-1, 7
Course Element (Video)	Gabrieli. *Operant Conditioning, Thorndike’s Cat, and Little Albert | Learning . . .*	MIT OpenCourseWare	CC BY-NC-SA	B-3, 4^b^, 5^a^, 8^a^, 9^a^
Chapter	Bouton. *Conditioning and Learning*	OER Commons	CC BY-NC-SA	B-3, 4^a,b^, 6^a^, 10
Chapter	Diener & Biswas-Diener (Eds). *Discover Psychology 2.0 – A Brief Introductory Text*	BC Campus: Open Ed	CC BY-NC-SA	B-3, 4^a,b^, 6^a^, 10
Chapter	Gabrieli. *Discussion: Learning | Learning | Introduction to Psychology | Brain . . .*	MIT OpenCourseWare	CC BY-NC-SA	B-3, 4^b^
Chapter	Stangor. *Introduction to Psychology*	BC Campus: Open Ed	CC BY	B-3, 4^b^, 5^b^, 6^b^, 8^a^, 11^a^ G-7
Chapter	Walinga & Stangor. *Introduction to Psychology – 1st Canadian Edition*	BC Campus: Open Ed	CC BY-NC-SA	B-3, 4^b^, 5^b^, 6^b^, 8^a^, 11^a^ G-7
Chapter	Biswas-Diener. *Introduction to Psychology: The Full Noba Collection*	Merlot.org	CC BY	B-3, 4^a,b^, 6^a^, 10
Chapter	Little et al. *Introduction to Sociology – 2nd Canadian Edition*	BC Campus: Open Ed	CC BY-NC-SA	
Chapter	Gabrieli. *Lecture 9: Learning*	MIT OpenCourseWare	CC BY	B-3, 4^b^, 5^a^, 6^b^, 8^a^
Chapter	Spielman et al. *Operant Conditioning*	OpenStax CNX	CC BY-NC-SA	B-3, 4^b^, 5^b^, 6^b^, 8^a^ G-7, 17
Chapter	Stangor. *Principles of Social Psychology*	Open Textbook Library	CC BY-NC-SA	B-3, 4^b^, 6^b^,
Chapter	Jhangiani et al. *Principles of Social Psychology – 1st International Edition*	BC Campus: Open Ed	CC BY	B-3, 4^b^, 6^b^,
Chapter	Lancombe et al. *Psychology*	OER Commons	CC BY-NC-SA	B-3, 4^b^, 5^b^, 6^b^, 8^a^ G-7, 17
Chapter	Biswas-Diener. *Psychology as a Biological Science*	Open Textbook Library	CC BY-NC	B-3, 4^a,b^, 6^a^, 10
Chapter	Spielman et al. *Psychology, Learning, Introduction*	OER Commons	CC BY-NCCC BY-NC	
Chapter	Spielman et al. *Psychology, Learning, What Is Learning?*	OER Commons	CC BY-NC-SA	B-3
Chapter	Spielman et al. *Psychology: OpenStax*	BC Campus: Open Ed	CC BY-NC-SA	B-3, 4^b^, 5^b^, 6^b^, 8^a^ G-7, 17
Chapter	Price. *Research Methods in Psychology*	BC Campus: Open Ed	CC BY-NC-SA	D-1, 2, 3^a^, 4, 5^a^
Chapter	Jhangiani et al. *Research Methods in Psychology – 2nd Canadian Edition*	BC Campus: Open Ed	*©*	D-1, 2, 3^a^, 4, 5^a^
Chapter	Price et al. *Research Methods in Psychology – 3rd American Edition*	Open Textbook Library	CC BY	D-1, 2, 3^a^, 4, 5^a^
Chapter	Kohn. *Skinner-Boxed*	MIT OpenCourseWare	©	B-4^b^
Chapter	Crocker et al. *Supporting Individuals With Intellectual Disabilities and Mental Illness*	BC Campus: Open Ed	©	B-9^a^ C-1^a^ F-6, 7^a^ G-14^a^ H-4
Reference	Sarafino & Graham. *Behavior Modification*	MIT OpenCourseWare	©	
Reference	Todd. *FBA Matrix: A National Survey of the Effectiveness of a Functional Behavior Assessment Matrix in Generating Existing Interventions*	Digital Public Library of America	CC BY-NC-SA	
Reference	Fitzsimmons. *Functional Behavior Assessment and Behavior Intervention Plans*	Digital Public Library of America	Unknown	F-2^b^, 4, 6^b^, 7^b^, 8^b^, 9^b^
Reference	Krasnegor. *Behavioral Analysis and Treatment of Substance Abuse*	HathiTrust - Full View	©	
Reference	Congressional Research Service: Richard N. Apling, Domestic Social Policy Division; and Nancy Lee Jones, American Law Division. *Individuals With Disabilities Education Act (IDEA) – MIT*	MIT OpenCourseWare	©	
Reference	Shors & Matzel. *Long-Term Potentiation: What’s Learning Got to Do With It?*	MIT OpenCourseWare	©	
Reference	Shabani et al. 2002. *MAS 962: Autism Theory and Technology*	MIT OpenCourseWare	CC BY-NC-ND	
Web	Autism Speaks. *ABA Therapy*	Merlot.Org	©	B-4^a^
Web	Scherer/B. F. Skinner Foundation. *B. F. Skinner*	Merlot.Org	©	
Web	Madden. *Division 25 – Division of Behavior Analysis*	Merlot.Org	CC BY-SA	
Web	Kersley. *Explorations in Learning and Instruction: The Theory Into Practice Debate*	Merlot.Org	©	B-1^a,b^ B-2^a,b^ B-4^a,b^
Web	Iris Center. *Functional Behavioral Assessment: Identifying the Reasons for Problem Behavior and Developing a Behavior Plan*	OER Commons	CC BY-NC-ND	B-11^a^ C-1, 8 F-3^a^, 6, 7 H-1, 8, 9
Web	HOBA SIG. *History of Behavior Analysis*	Merlot.Org	©	
Web	Miller. *Multimodal Functional Behavioral Assessment Behavior Intervention Plans*	Merlot.Org	©	F-7
Web	Plaud. *The Cambridge Center for Behavioral Studies*	Merlot.Org	©	

OERs were recovered using MOM. Resources are sorted according to type of information (column 1), OER repository (column 3), copyright license (column 4), and relevance to the BACB task list (5^th^ ed., column 5). Note that author and title information (column 2) does not conform to a metadata standard but are reported as the resource appears in the repository metadata record to facilitate resource recovery

^a^Resources including information about a task list item but lacking depth or breadth

^b^Resources presenting information that is inaccurate

The largest category of materials found were coded as book chapters (*n =* 22, 35.5%), followed by course elements (*n =* 16, 25.8%), websites (*n =* 8, 12.9%), references (*n =* 8, 12.9%), applications (*n =* 4, 6.5%), textbooks (*n =* 3, 4.8%), and course builds (*n =* 1, 1.6%). Unfortunately, nine records representing two applications, one chapter, three course elements, and a course build could not be found via MOM search or through a search of the referenced repository, suggesting that they had been removed from the originating repository’s index. Because these resources could not be further analyzed, they were omitted from any subsequent analyses. Table 2 lists the remaining 55 items included for subsequent analyses.

#### License

To help readers better understand if and how each resource found could be used, the 55 resources were reviewed to report what license, if any, was designated to that resource. Eighteen resources (32.7%) used traditional “all rights reserved” copyright, thirty-three resources (60.0%) used some variant of the public CC license, and one resource (1.8%) was in the public domain. Three resources (5.4%) included no information about license or permissions and were coded as “unknown” (Table 2).

#### Content

To better facilitate the efficient review of resources, each of the 55 resources was reviewed to determine if the content could be applied to teach necessary content knowledge to developing behavior analysts. Resource content was analyzed and compared to the Behavior Analyst Certification Board’s task list (5th ed., BACB, 2017). If the resource content appeared related to a specific subdomain, the resource was coded with the corresponding task list subdomain. For instance, if a resource included information about schedules of reinforcement, the resource was coded “B-5” to indicate that content corresponded to task list item B-5, which reads “define and provide examples of schedules of reinforcement” (BACB, 2017, p. 2). Resources including information about multiple task list items were scored accordingly by listing each task list item included (see column “BACB 5th Ed. Task List Designation”).

The reviewed resources varied in content and quality. Some resources appeared to provide some, but not all, of the necessary task list content items (e.g., the resource described positive reinforcement without reference to negative reinforcement). Other resources provided only a cursory examination of the content area, lacking the necessary depth to teach a modest understanding of the corresponding principle (e.g., positive reinforcement described as a procedure where a stimulus is added following behavior with no mention of any strengthening or increased rate of behavior). When resources lacked breadth or depth of coverage for a specific task list item, that task list code was subcoded with a superscripted “a.” Readers may infer that these resources could be a good start to creating more refined educational material but would be insufficient to address that task list item without revision.

Still other resources provided descriptions that appeared conceptually inaccurate or misleading (e.g., conflating the procedure of reinforcement with the delivery of a reward stimulus regardless of the impact on the rate or strength of a response; fixed-time schedules described as fixed-interval schedules). The task list code for these resources was subcoded with a superscripted “b” to denote that the content should be evaluated more stringently and revised before use as an educational resource.

### Coding Training

To determine whether the coding protocol could be consistently applied, the author and a research assistant independently coded search results using the previous categories. Given the unique search results returned by MOM, consistency of the coding protocol was evaluated by comparing codes applied to the list of 64 resources generated following the combination of all search results (described previously). Agreement was defined as both observers independently scoring each resource using the same category code, calculated as the number of agreements divided by the number of agreements plus disagreements and multiplied by 100 to create a percentage. Agreement on item coding was 90.5%. Subsequent analysis showed that most disagreement occurred when the metadata record was incorrect (e.g., the metadata-indicated reference was a website but actually linked to a book chapter).

## Search Results

### Available Behavior Analysis OERs

For the purposes of finding materials to supplement or create a course for new behavior analysts, it appears that OERs with behavior-analytic content do exist. However, most resources discovered in this review appear to primarily focus on teaching a brief module on learning theory in an introductory-level psychology survey course (*n =* 36, 63.1%). Subsequent content analysis showed that many of these resources either underexplain or incorrectly present behavior-analytic principles (see Table 2). These results seem consistent with previous research about the accuracy of behavior-analytic content in introductory psychology textbooks (Todd & Morris, 1983, 1992). Future studies may wish to systematically evaluate these new OER psychology textbooks to determine whether they share the same misrepresentations as earlier commercial textbooks. Furthermore, the CC license on these textbooks also means that interested parties may choose to revise and redistribute these resources, publicly correcting misinformation about the science of behavior analysis (Morris, 1985).

 The review revealed two promising full-length books written specifically on topics adjacent to behavior analysis. Sennott et al.’s *Comprehensive Individualized Curriculum and Instructional Design* (2015) appears to be written to supplement a graduate-level course in special education. Desrochers and Fallon’s *Instruction in Functional Assessment* (2014) was written to “provide instruction in [functional assessment] skills for pre-professionals in the fields of education and psychology” (p. 4). Both books provide an example of how high-quality OERs may be written and effectively distributed to a wider audience.

### A Note About Licenses

Most works returned were shared under some variation of a CC license (e.g., some rights permitted). However, some resources discovered through MOM appeared to be fully copyrighted (“all rights reserved”/©). This raises some concern, and the reader should not assume that inclusion in an OER repository database ensures permissions to retain, edit, or redistribute. Users should carefully review all resources for licensing information and become familiar with the variety of copyright licenses to better understand what rights and permissions, if any, are given for each resource. These results also suggest that content creators do not always clearly communicate what permissions, if any, they share with their audience, highlighting the need for creators to include information about usage rights with their resources.

## Discussion

### Benefits of More Behavior Analysis OERs

The obvious benefit of OERs is their cost. If the aim of adopting OERs is simply to save students money, it would be easy enough for instructors to adopt materials that are shared under a traditional copyright license yet are free to access for the user, such as Ted Talks, YouTube videos, or materials created by content creators like The Daily BA (n.d.) or BehaviorBabe (2019). However, these materials are nevertheless limited in their utility for course instructors. These resources cannot be retained, edited, or redistributed. Course instructors would be at the liberty of content creators to continue using those resources.

For example, imagine a set of course resources that includes assigned YouTube videos created by someone other than the course instructor. According to YouTube’s terms of service, users cannot download any content unless there is an explicit tool to do so provided by and sanctioned by their service, and users cannot copy, redistribute, or transmit content without the express written permission of YouTube or the licensors (YouTube, 2018). Should the video be taken down, moved, or changed in any way, instructors could lose access to that resource indefinitely. An instructor could never truly rely on that video content being available for a course without the right to download, retain, and redistribute the content to students without violating the YouTube terms of service. This would apply to all other excellent, free-to-access materials shared in our field. As a result, no durable course build can reasonably evolve from free-to-access resources shared under a traditional copyright license.

The rights associated with a CC license—including free access, indefinite retention, and redistribution—confer additional advantages to instructors and students beyond cost savings (Baraniuk, Finkbeiner, Harris, Nicholson, & Williamson, 2017). Materials that can be edited by a larger community will evolve in quality and can be easily edited or remixed to meet changes in educational standards, course sequence requirements, or unique learner needs (Inclusive Design Research Center, n.d.). Naturally, the quality of these edits will be largely dependent on factors such as the quality of the research and robust peer review surrounding those works, the ability of editors to refine the content, and the medium through which the content is delivered. Improvements in self-publishing software (e.g., Pressbooks, 2019) and peer-review networks such as those provided by the Open Textbook Network (2018) have facilitated the growth of a robust alternative textbook-publishing market.

Educational materials shared under a CC license also permit the larger community to develop derivative works, such as essential ancillary educational resources. OERs also hold the promise to increase dissemination of behavior analysis and offer an opportunity to create a variety of low- or no-cost training and reference materials (e.g., study materials, infographics, task analyses, walkthrough guides) aligned with course outcomes to meet students’ educational needs.

Finally, more educational materials shared under a CC license can help foster conversations around intellectual property and permissions. Section 8.02a of the *Professional and Ethical Compliance Code for Behavior Analysts* (BACB, 2014) clearly specifies the need to honor intellectual property by seeking permission to use trademarked material, as well as to clearly communicate rights and permissions on materials. However, the language of our ethics code tacitly speaks only to fully copyrighted materials; there is no reference to or guidance on works shared under a CC license or materials found in the public domain. For instance, materials shared under a CC BY-NC-SA license would not require the licensee to obtain permission to use the material prior to adaptation but would require the licensee to share derivative works under a similar or less restrictive license. By failing to account for the variety of available copyright licenses, our code of ethics presents the issue of intellectual property from a very black-and-white perspective and may fail to account for the many ways that an author may choose to share work.

Given the complexity of copyright, fair use exemptions, and public copyright licenses, it seems like time for a more nuanced conversation around intellectual property rights and permissions in the education and practice of behavior analysis. Concerns about ridicule from the community, the potential for inconsistent application, and authors’ concerns about the misuse of their content are all barriers to the creation of freely shared material in our field. CC licenses can help address some of these concerns via the wide range of permissions and restrictions they provide. For instance, the permission to redistribute and use but not edit or change content is provided by licenses containing the ND feature. This license might be ideal for authors who are concerned about maintaining the fidelity of their content. Wider use of the CC license may set the occasion for our field to reaffirm a commitment honoring the wishes of the author, creating an environment where authors will have greater confidence that their materials will be used in the manner they intend.

Educational materials shared under a CC license also permits OER-enabled pedagogical practices (OEPs), allowing students to become content creators. For example, past student-focused OEP projects have included developing a literature anthology (DeRosa, 2015), creating ancillary course materials for an open textbook (Jhangiani, 2017), and authoring a peer-reviewed article (Madan, Ferrara, & Lev, 2017). Student-created OEPs in behavior analysis could produce several benefits for the field. First, students as creators can help increase the amount and variety of OER materials available in behavior analysis. According to BACB CEO Dr. James Carr, the field of behavior analysis will have a significant shortage of educational content as authors of today’s behavior analysis textbooks retire and those books go out of print (Cicoria, 2018).

For instance, instructors could teach students to create educational materials to evaluate whether students can accurately describe behavior-analytic content in a way that is both technologically precise and easy to understand for a novice audience. This ability to quickly and effectively switch to verbal behavior that best communicates content to the listener is of paramount importance for a field sometimes accused of having a communication problem (Bailey, 1991; Freedman, 2016), especially if using reader-preferred language yields improvements in treatment acceptance (Critchfield et al., 2017) and procedural fidelity (Jarmolowicz et al., 2008). The ability to explain behavioral concepts using both technical and nontechnical language is a critical skill for practitioners, referenced in both the fourth and fifth edition task lists (BACB, 2012, 2017).

Beyond simply creating a greater capacity for OER generation through student-created materials, students as authors could also fundamentally change the quality of our educational materials. The majority of textbooks in behavior analysis are written by authors from a single gender identity, sexual orientation, native language, and ethnicity. As a result, these educational materials will inherently reflect that narrower perspective as a function of the authors’ similar shared learning history. By creating a system where students can be content creators with the guidance and support of instructors, we could begin to see content that better reflects the diversity that the field of behavior analysis yearns to support (e.g., Nava, Fahmie, Jin, & Kumar, 2019).

### Promoting Open Content Creation

In order to understand why there are so few behavior-analytic OERs, one must first understand the contingencies supporting the creation and dissemination of open content. The two books identified in this review (Desrochers & Fallon, 2014; Sennott et al., 2015) were supported with grant funding provided by university open textbook initiatives. Although some universities have created programs to support OER adoption via financial compensation, course buyouts to free faculty time, and hiring support personnel to assist in the transition from commercial to OER materials, university open textbook initiatives remain the exception rather than the rule.

Support for OERs has been attempted on a larger scale as well. Variations of the Affordable College Textbook Act (ACTA) have been introduced to the US Senate three times since 2013 (Lieberman, 2018). The aim of this law is to make college textbooks more affordable, similar to how the 2008 Higher Education Opportunity Act reduced textbook costs by notifying students of textbook costs at registration and requiring textbook publishers to unbundle educational material (20 U.S.C. § 1015b, 2008). As of this writing, no version of ACTA has yet been passed through Congress. Thus, financial resources to support OER creation remain scarce, and a top-down approach to promoting OER creation may not be soon coming.

However, most educational materials are created by professors and university faculty. Focusing on only financial contingencies ignores other professional contingencies that may influence the decision to create OERs. As described by Reed (2014), pretenure faculty must prioritize the activities that will most efficiently help them secure promotion and tenure (P&T). P&T guidelines tend to be set by individual departments and universities and may differentially favor the values of those individual units (e.g., publishing in high-profile journals, engaging in teaching practices associated with student success, securing extramural grants). If the field of behavior analysis truly values diversity—and the adoption of OERs has been robustly demonstrated to improve student success, especially for students traditionally underrepresented in higher education—one clear way to foster the creation of OERs is for departments and universities to differentially favor the creation, evaluation, and dissemination of OERs in P&T guidelines.

However, these changes are unlikely to happen without some external pressure. Students of behavior analysis may begin by asking instructors or departments for open educational materials (Vitez, 2018), by modeling the use of open licensing by clearly indicating which licenses their intellectual property (e.g., course assignments) holds, and by appealing to their student university and Association for Behavior Analysis International (ABAI) representatives to raise awareness of and support for the adoption of OERs.

Likewise, faculty can be a strong force for effecting system change in P&T guidelines that would facilitate the adoption and creation of OERs. For example, faculty governance groups may collectively recommend highlighting OER adoption an evidence-based, high-impact pedagogical practice or OER creation as innovative creative/research activity (e.g., Frieden, 2018). Faculty serving as P&T committee members or external reviewers may highlight faculty adoption or creation of OERs in their recommendations, not only because of the beneficial impact that OERs have on student success described previously, but also because the adoption of OERs may require more time and effort compared to the adoption of equivalent commercial products (Bliss, Hilton, Wiley, & Thanos, 2013). Most work related to OERs, from creation to curation to adoption, is the product of voluntary contributions. Faculty adopting OERs often do so because of the direct benefits to students, even if it comes at a direct cost to the productivity of the faculty.

Likewise, professional organizations such as ABAI may be an effective force for change. ABAI currently honors contributions to the field ranging from distinguished service to dissemination via mass media or to international consumers (ABAI, 2019). Perhaps the adoption and creation of OERs could be highlighted through one of these awards or through a new award dedicated to effective or inclusive teaching. Alternatively, ABAI may support the creation of OERs through creator grants.

### Disseminating OERs and Concerns With Metadata

This study included several separate stages, ranging from the initial search for resources through coding for specific content. Throughout these stages, some but not all resources discovered during earlier searches were recovered for later analysis. This lack of reliable access across the span of this review demonstrates unique challenges for the longevity of resources facing the OER community, especially when compared to alternative forms of searching (i.e., literature review).

Traditional literature reviews (e.g., Alvero, Bucklin, & Austin, 2001; Vladescu & Kodak, 2010) are only possible because of the quality of the indexed metadata found in most major databases. When articles are accepted and committed to print, they are indexed in a database and a durable record of the item and relevant metadata (e.g., author, year of publication, abstract, page numbers) are generated and managed by a trained specialist (e.g., librarian, data scientist, cataloger). The reproducibility of these reviews is dependent on the high quality of that data, and anyone who has access to those journals should be able to use the same methodology and return the same results.

This review highlights the challenges with the metadata of OERs. A replication of the method used for this review would likely not reproduce precisely the same search results for several reasons. First, there is no metadata standard used across the repositories targeted by MOM. Some repositories are more firmly managed, and the metadata are generated by a highly trained professional (e.g., a librarian), increasing the likelihood that the data will be accurate and specific. In other repositories, the metadata are generated by volunteer users or by computer scripts designed to scrape the resource and return a full text-based metadata record. These methods result in metadata that may be inaccurate or may omit important information, such as title, author, or permissions and copyright. Due to the inconsistency in metadata quality between resources and repositories, the resources returned by MOM for this search varied significantly in accuracy. There were duplicate resources, resources with incorrect titles or authors, and many materials in the repositories that are not truly OERs. These errors have been preserved in Table 2 to permit the reader to search MOM and locate the resources (if still available), but this highlights the need for greater financial support for OER repositories, as well as the value of a more consistent metadata standard across repositories.

Second, unlike popular web search engines, MOM has no preindexed search results and has a limited range of locations to search. When a query is entered, real-time search results are returned (as described previously). If the index of one repository has changed (e.g., if a resource is no longer available or if the metadata around that resource have been altered), the search results returned to MOM may be different. The results returned to MOM are also limited to only those results found in the linked search engines. OERs located in other web locations, including the rich body of videos shared on YouTube using an open license (e.g., PsychCore) did not return as part of the search. The extent to whether an OER was discovered in this search was dependent on whether that resource had been added to an OER repository. OER creators that host their content on other sites (e.g., YouTube) may be well advised to add a record of their work to an OER repository (e.g., Merlot, Creative Commons) to increase discoverability. The limitation of finding OERs across a wide range of sites may need to be addressed through future technological advances, and future studies may wish to explore how to reduce barriers to finding OERs as a way of better understanding what other OERs are available for use.

Last, because of the inconsistency in metadata between repositories, MOM searches the entire metadata record for the search term. As a result of this search approach, many results returned to the user may bear little resemblance to the search terms entered (i.e., many “false positive” resources). MOM casts a wide net and sacrifices specificity for returning the maximum number of search results. Although this yields the largest number of possible resources, it also requires that the user review each result to determine the relevance to the initial search aims. Alternatively, users may search specific repositories for resources, but the opposite effect will occur: Without knowledge of the specific, narrow terms used by the metadata creator, the user may find few resources (i.e., false negatives) during their searches, suggesting that few resources are available.

These points should not be interpreted as a criticism of the metafinder tool. Because the initial review of OERs was conducted in fall 2018, MOM now references two new repositories and has increased the number of available search fields, now permitting users to search by title and author in addition to the full record. Users can also set an alert for specific search terms and be notified when new resources become available. Although additional features, such as the ability to change the number of search results displayed or export search results, would be useful tools, this author believes that MOM remains the most robust tool available to efficiently search for relevant OERs in a wide variety of sources. Users interested in finding OERs should also conduct lateral searches across many search engines and, if possible, partner with librarians and library faculty who specialize in OER content to find available resources.

## Conclusion

Behavior analysts have previously called for the free and open distribution of research materials through an open license as an issue of social justice (Mattaini, 2004), but our field has yet to formally call for the free and open distribution of educational materials (Pavone, 2018). It cannot be understated that the high cost of educational materials is a barrier to student success. Research shows that students regularly forgo purchasing their textbooks due to cost (Florida Virtual Campus, 2012, 2016) or attempt to succeed without course materials, resulting in poorer grades, more student course withdrawals, and more failing grades (Fischer et al., 2015). When instructors use OERs, they do not simply save students money; faculty using OERs help students enroll in more classes, persist through courses, and ultimately graduate from college (Hilton, Fischer, Wiley, & Williams, 2016; Jhangiani & DeRosa, 2017).

OERs help support all students by ensuring they have access to course materials from the first day of classes (Hilton, 2016; cf. Grimaldi et al., 2019), with the largest benefits for students traditionally underrepresented in higher education (Colvard et al., 2018). When as little as $300 can make the difference between a student persisting in college and dropping out (GSU, n.d.), the cost of our textbooks and a lack of behavior analysis OERs maintain systemic inequities in the field of behavior analysis.

If first-generation, low-income, and ethnic minority students cannot persist and graduate because of the cost of textbooks, how can they hope to become future behavior analysts? If the field of behavior analysis values diversity and inclusion, access to high-quality and low-cost educational materials must be part of the solution. This review of available behavior analysis OERs suggests there simply are not enough resources currently available to reach this goal. Although OERs may not address all issues of educational access, beginning with an open textbook on the principles of behavior analysis could be enough to start a sea change. It is at least a place to start.
